# Plasminogen mediates communication between the peripheral and central immune systems during systemic immune challenge with lipopolysaccharide

**DOI:** 10.1186/s12974-019-1560-y

**Published:** 2019-08-28

**Authors:** Sarah K. Baker, Zu-Lin Chen, Erin H. Norris, Sidney Strickland

**Affiliations:** Patricia and John Rosenwald Laboratory of Neurobiology and Genetics, The Rockefeller University, New York, NY 10065 USA

**Keywords:** Plasminogen, Lipopolysaccharide, Perivascular macrophages

## Abstract

**Background:**

Systemic inflammation has been implicated in the progression of many neurodegenerative diseases and may be an important driver of the disease. Dementia and cognitive decline progress more rapidly following acute systemic infection, and systemic inflammation midlife is predictive of the degree of cognitive decline. Plasmin, the active form of the serine protease plasminogen (PLG), is a blood protein that plays physiological roles in fibrinolysis, wound healing, cell signaling, extracellular matrix degradation, and inflammatory regulation.

**Methods:**

Mice were treated with an antisense oligonucleotide to deplete liver-produced PLG prior to systemic challenge with lipopolysaccharide (LPS), a major component of the outer membrane of gram-negative bacteria, known to induce a strong immune response in animals. Following treatment, the innate immune response in the brains of these animals was examined.

**Results:**

Mice that were PLG-deficient had dramatically reduced microgliosis and astrogliosis in their brains after LPS injection. We found that blood PLG regulates the brain’s innate immune response to systemic inflammatory signaling, affecting the migration of perivascular macrophages into the brain after challenge with LPS.

**Conclusions:**

Depletion of plasma PLG with an antisense oligonucleotide dramatically reduced glial cell activation and perivascular macrophage migration into the brain following LPS injection. This study suggests a critical role for PLG in mediating communication between systemic inflammatory mediators and the brain.

## Background

Systemic inflammation may play a role in the development of a vast array of neurological disorders and diseases. However, exactly how systemic inflammatory signals influence the neuroimmune response, either directly or indirectly, is not well understood. Communication of the peripheral immune system with the brain is evidenced by the fact that individuals are at increased risk for dementia following systemic infections such as influenza or ulcerative colitis [1].

Recent results from the Atherosclerosis Risk in Communities study, which included over 12,000 participants, suggest that systemic inflammation in midlife is predictive of 20-year cognitive decline. Individuals with the highest levels of systemic inflammation in midlife had the steepest cognitive decline in the 20-year period analyzed, especially in terms of memory, suggesting that inflammation may be a driver of cognitive decline years later [2].

Lipopolysaccharide (LPS) injections have long been used as a mouse model of systemic inflammation [3]. LPS is a gram-negative bacterial toxin that causes activation of the Toll-like receptor 4, which is expressed on myeloid cells including monocytes, macrophages, granulocytes, and dendritic cells [4]. LPS is a good model to study the connection between peripheral and central immune responses; when injected systemically, LPS leads to the activation of microglia, the major immune cells of the brain, which then release proinflammatory signals into the parenchyma [5].

Plasminogen (PLG) and its active form, serine protease plasmin, are blood proteins that play roles in fibrinolysis, wound healing, cell signaling, extracellular matrix degradation, and inflammatory regulation [6]. Using a mouse model of Alzheimer’s disease (AD), we recently showed that plasma PLG deficiency is protective against AD pathology and drastically decreases the brain’s innate immune response. We also showed that driving up plasmin activity exacerbates this response [7]. In this case, amyloid beta, a protein found deposited in the brains of AD patients, was genetically introduced to induce an inflammatory response in the mouse brain, but depleting PLG only in the periphery caused a decrease in this neuroimmune response, leading to an important finding about the regulation of neuroinflammation from systemic molecules.

Due to the link between systemic inflammation and cognitive decline, we were interested to determine whether PLG plays a key role in the regulation of central nervous system (CNS) inflammation in cases of a systemic inflammatory challenge. In this study, we depleted PLG peripherally in mice and then challenged the animals with LPS injected into the intraperitoneal cavity. We analyzed the brains of these animals to determine any effect on the CNS immune response.

## Methods

### Animals and ASO treatment

All animal experiments were conducted in accordance with the guidelines of the US NIH Guide for the Care and Use of Laboratory Animals and with approval from the Animal Care and Use Committee of The Rockefeller University. C57Bl/6 mice (Jackson Laboratories) were used for all experiments. Antisense oligonucleotides (ASOs) against PLG and a scrambled control (CTRL) from Ionis Pharmaceuticals are 20 nucleotides long, target the liver, and are chemically modified with a phosphorothioate backbone and 2-*O*-methoxyethyl wings for stabilization and to optimize the efficiency of the knockdown. ASOs are not predicted to have off-target effects (genomic targets or non-genomic proinflammatory effects). CTRL or PLG ASO-treated mice were administered either LPS or phosphate-buffered saline (PBS) such that there were four cohorts of mice used in this experiment: CTRL ASO-PBS, PLG ASO-PBS, CTRL ASO-LPS, and PLG ASO-LPS (*n* = 5–7 mice/group). Mice were treated at a dose of 150 mg/kg/week for 2 weeks starting at 10 weeks of age.

### LPS injections

Lipopolysaccharides from *Escherichia coli* O111:B4 (Sigma) were injected into the intraperitoneal cavity of mice at a dose of 1 mg/kg for three consecutive days to robustly activate microglia. Mice were anesthetized and perfused with saline 4 h following the final LPS injection.

### Intraperitoneal macrophage collection and staining

Intraperitoneal (IP) macrophages were collected from CTRL ASO- and PLG ASO-treated mice by injecting 15 mL PBS into the peritoneal cavity and aspirating the fluid using a 31-gauge needle. Cells were then spun down, collected, and plated. After growing for 1 week, cells were fixed in 4% paraformaldehyde and stained with PLG-R_KT_ (Epigentek), CD11b (DSHB), and DAPI (Vector Laboratories) and imaged on an Echo Revolve Upright Epifluorescent microscope at × 20.

### Plasma preparation

At the time of anesthesia, prior to perfusion, blood was collected by retroorbital plexus bleeding, and plasma was isolated as described previously [7].

### Western blotting

Plasma samples were run on reducing SDS-PAGE gels, transferred to PVDF membrane (BioRad) on a Trans-Blot Turbo Transfer System (BioRad), incubated overnight at 4 °C in primary antibody (rabbit anti-PLG, Abcam; rabbit anti-transferrin, Abcam), and then incubated with an appropriate HRP-conjugated secondary antibody. Blots were developed with a chemoluminescent substrate (BioRad) and visualized on a ChemiDoc Imaging System (BioRad).

### Whole blood staining

At the time of anesthesia, prior to perfusion, blood was collected by retroorbital plexus bleeding. Blood smears from each animal were created on slides and immediately frozen prior to staining. Slides were fixed in 4% paraformaldehyde and stained with PLG-R_KT_ (Epigentek), CD11b (DSHB), and DAPI (Vector Laboratories) to detect PLG-R_KT_ expression of leukocytes (CD11b+ cells).

### Immunohistochemistry

Mice were deeply anesthetized and perfused with saline prior to brain collection. Brains were immediately frozen for brain sectioning and immunohistochemical analysis. Sections were fixed in methanol to acetone (1:1). Primary antibodies used were against CD11b (microglia/macrophages, DSHB), CD68 (microglia/macrophages, BioRad), glial fibrillary acidic protein (GFAP; astrocytes, Dako), PLG-R_KT_ (Epigentek), CD206 (perivascular macrophages, Invitrogen), and collagen IV (blood vessels, Millipore). After incubation in the primary antibody for 3 h at room temperature, brain sections were rinsed in PBS and then incubated with a fluorescent dye-conjugated secondary antibody for 1 h. Brain sections were washed with PBS and coverslipped with fluorescence mounting media (Vectashield).

### Imaging analysis

Following immunostaining, brain sections were imaged with a Nikon Eclipse Ti2-U microscope equipped with Appo Fluor (× 4 NA 0.2, × 20 NA 0.75, × 40 NA 0.6) objectives at room temperature using air as the imaging medium and a Qi2 monochrome camera. Nikon Elements software was used to acquire images from a camera and to analyze the percent area for each staining. A researcher blind to the treatment of each mouse analyzed the total area of positive staining as a percentage of total image area (*n* = 3–4 sections/mouse; 5–7 mice per group).

### Statistical analysis

Statistical analyses were conducted using GraphPad Prism software for two-way analysis of variance (ANOVA), as indicated in each figure legend. All values presented in graphs are mean ± SEM.

## Results

### An ASO directed against liver-produced PLG is sufficient to knockdown PLG levels in the plasma of mice, and monocytic cells collected from the blood and peritoneal cavity of mice express PLG-R_KT_, a PLG receptor important for chemotaxis

**Fig. 1:**
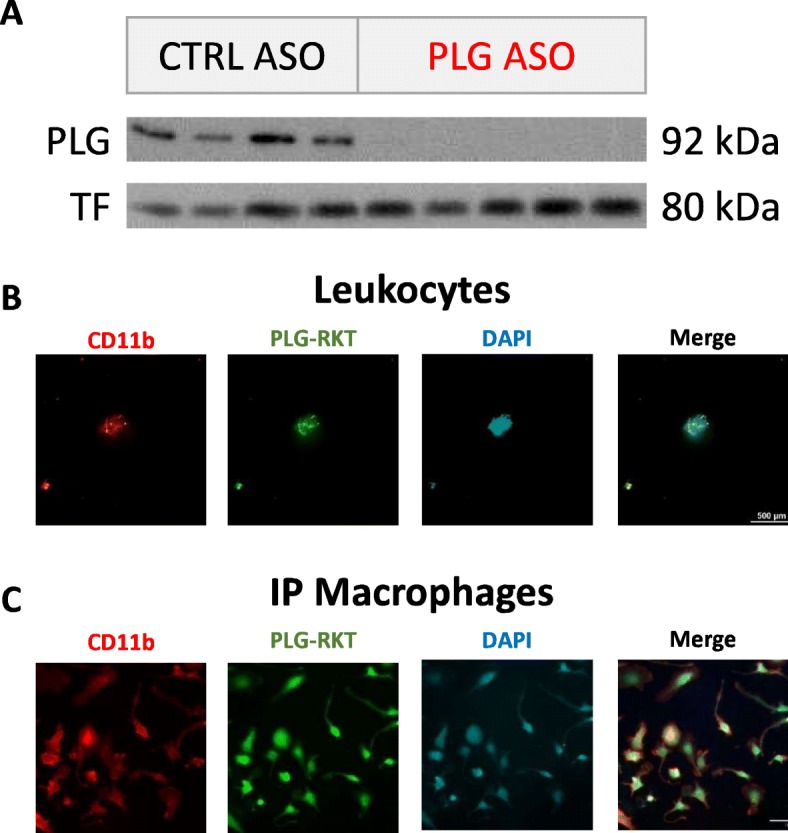
PLG is depleted in the plasma of PLG ASO-treated animals and many monocytic cells throughout the body express PLG-R_KT_, the purported receptor responsible for PLG-mediated chemotactic migration. **a** Representative Western blot of PLG and transferrin (TF) levels in the plasma of CTRL ASO- and PLG ASO-treated mice. PLG-R_KT_ is expressed **b** on leukocytes (CD11b+ cells, red) in the blood and **c** on IP macrophages (CD11b+ cells, red) isolated from the peritoneal cavity. PLG-R_KT_ expression is also observed on perivascular macrophages (CD206+ cells, red) in the brains of these mice (Fig. 3b of the manuscript). Scale bar, 100 μm. *n* = 3 sections/brain, 5–7 mice/group

To investigate the role of plasminogen in the interaction between the periphery and the brain during the innate immune response, we used an ASO-mediated gene knockdown strategy to deplete liver-produced PLG (PLG ASO) in the plasma of mice. Control mice were administered a scrambled control ASO (CTRL ASO). PLG level was depleted in the plasma of PLG ASO-treated mice compared to that of CTRL ASO-treated mice (Fig. 1a). We examined the expression of PLG-R_KT_, the purported receptor responsible for PLG-mediated cell migration, and found that it is highly expressed in leukocytes circulating in the blood (Fig. 1b) and intraperitoneal (IP) macrophages (Fig. 1c).

### Plasma PLG depletion dramatically decreases microglial and astrocytic cell responses to systemic LPS challenge

**Fig. 2:**
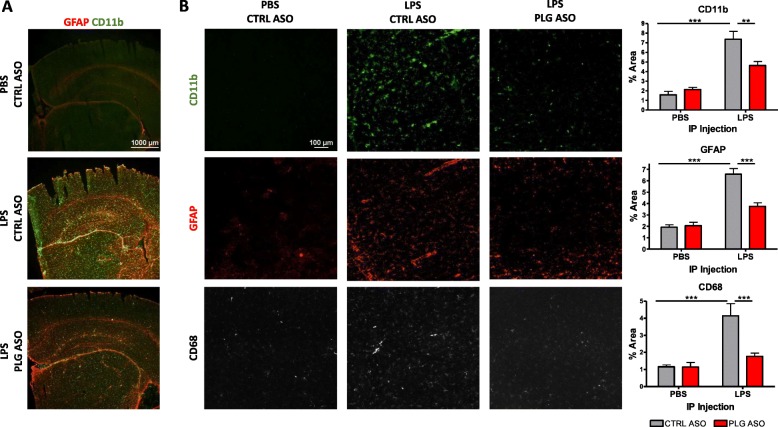
Glial cell activation is reduced in the brains of PLG-depleted mice following injection with LPS. **a** GFAP (red) and CD11b (green) staining in PBS-CTRL ASO, LPS-CTRL ASO, and LPS-PLG ASO mouse brains. Scale bar, 1000 μm. *n* = 4 sections/brain, 5–7 mice/group. **b** GFAP (red), CD11b (green), and CD68 (white) staining and quantification in the cortex of CTRL and PLG ASO-treated mice injected with PBS or LPS (× 20 magnification). PBS-injected PLG ASO images are not shown because they are indistinguishable from those of PBS-injected CTRL ASO. CD11b, GFAP, and CD68 expression levels are increased significantly in LPS-treated animals compared to PBS-treated animals following CTRL ASO injection (***p < 0.001 for all comparisons). CD11b (**p < 0.01), GFAP (***p < 0.001), and CD68 (***p < 0.001) are decreased significantly in LPS-injected PLG-deficient animals compared to LPS-injected CTRL ASO mice. Scale bar, 100 μm. *n* = 4 sections/brain, 5–7 mice/group

When exposed to systemic LPS on three consecutive days, mice showed a global increase in CD11b (microglia/macrophages) and GFAP (astrocytes) expression throughout whole coronal brain sections (Fig. 2a), indicating the activation of the brain’s resident microglia and astrocytes, and possible migration of peripheral macrophages into the brain. More specifically in the cortex, LPS injection robustly induced expression of astrocyte marker GFAP (~ 3-fold increase), as well as two microglial/macrophage markers, CD11b and CD68 (~ 4-fold increase for both) (Fig. 2b).

Remarkably, peripheral PLG depletion led to a markedly decreased immune response by astrocytes and microglia/macrophages in the brain after LPS challenge (Fig. 2a, b).

### Plasma PLG depletion decreases perivascular macrophage migration to the brain during systemic LPS infection, possibly via the PLG-R_KT_ receptor

**Fig. 3:**
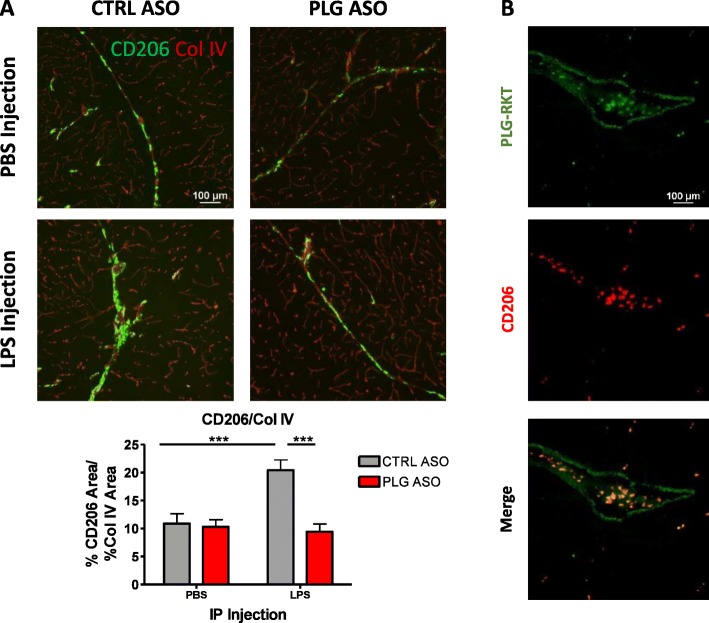
PVM accumulation in the brain increases with LPS challenge and is mediated by plasma PLG. **a** Representative images and quantification of PVMs (CD206, green) in blood vessels of the brain (Col IV, red) in CTRL or PLG ASO-treated mice challenged with LPS or PBS. CD206 staining increases with LPS injection (*p* < 0.001) in CTRL ASO mouse brains but returns to control levels in PLG-deficient mice challenged with LPS (*p* < 0.001). **b** PLG-R_KT_ (green) is expressed on PVMs (red, CD206+ cells) in the brains of these animals and could be aiding in the migration of PVMs into the brain during LPS challenge. Scale bar, 100 μm. *n* = 3 sections/brain, 5–7 mice/group

We analyzed whether a difference in immune response between CTRL ASO and PLG ASO animals treated with LPS could be attributed to a decrease in perivascular macrophage (PVM) migration in PLG-deficient animals. Therefore, we examined the expression of CD206, a marker of PVMs, around brain blood vessels of CTRL or PLG ASO animals injected with either LPS or PBS (Fig. 3a). PVM staining was observed in major vessels of the brain that contain a perivascular space, and PVM accumulation in these spaces increased with LPS injection. Brains of LPS-injected PLG ASO mice had significantly reduced CD206 expression levels compared to that of LPS-injected CTRL ASO animals, similar to the expression level found in PBS-injected CTRL ASO mice (Fig. 3a). We examined the expression of PLG-R_KT_, the purported receptor responsible for PLG-mediated cell migration on CD206+ cells, and found that it is highly expressed on PVMs (Fig. 3b). Since PVMs normally express PLG-R_KT_, which binds to PLG during chemotactic migration of monocytic cells, the lack of systemic PLG in PLG ASO mice likely inhibits the migration of PVMs into the brain during a systemic challenge with LPS.

## Discussion

Here, we show that PLG plays a crucial role in regulating the neuroimmune response to a peripheral immune challenge with LPS. We have previously reported that plasmin, the active serine protease form of PLG, is likely responsible for the regulation of this activity, and have shown that treatment with PLG ASO is specific in knocking down plasma levels of PLG without affecting brain expression of various proteins in the PLG activator pathway [7]. In the current study, we add evidence that plasmin is a key modulator of communication between the nervous system and the immune system.

Activation of the innate immune system is a crucial step for the body when battling pathogens. However, when this activation becomes chronic, it can have detrimental effects. Microglia are the brain’s primary immune cells that are continually surveying their surroundings to release other inflammatory mediators or to phagocytose cells and protein aggregates [8]. Chronic microglial activation has been linked to neurodegeneration and clinical manifestations of dementia [9]. Furthermore, many of the risk factors for cognitive decline are associated with systemic inflammation, including obesity, hypertension, infection, cerebral infarction, smoking, and diabetes [1, 10–12].

As a broad-spectrum protease, PLG has many physiological functions, and it is understood that PLG interacts with cell surfaces for many of these roles. PLG, considered a proinflammatory cell activator, is a potent chemoattractant for monocytes, macrophages, and dendritic cells [13, 14]. There is also evidence that PLG plays a role in innate immunity by regulating macrophage phagocytosis [15] and can affect inflammatory cell function through the production of cytokines, reactive oxygen species, and other inflammatory mediators [16]. Previous work in our lab has shown that PLG^−/−^ mice have a decreased neuroimmune response following hippocampal injection of LPS [17]. In this study, however, there was no way to distinguish between the roles of brain- and liver-derived PLG in the regulation of this response. We extended this study by using an ASO that specifically targets liver-produced PLG and found that it is plasma PLG that is crucial to regulating this response. Furthermore, we found that systemic challenge with LPS is sufficient to drive a neuroinflammatory response, and direct injection into the brain is not required. This situation better recapitulates what happens physiologically in the body in response to pathogens.

PLG receptors are distributed broadly on many types of cells, including monocytes, macrophages, endothelial cells, and platelets [16]. The proteolytic functions of PLG allow it to degrade extracellular matrices and activate growth factors that aid in cell migration. Studies with PLG^−/−^ mice have shown that monocytes and macrophages have an impaired ability to migrate to the peritoneum when thioglycollate, an inducer of neutrophils, granulocytes, monocytes, and lymphocytes, is injected [18]. Furthermore, mice with increased plasmin activity due to knockout of alpha-2-antiplasmin, a plasmin inhibitor, have increased macrophage recruitment in the thioglycollate injection model [19]. Moreover, macrophage recruitment is suppressed when mice are given tranexamic acid, a lysine analog that inhibits PLG, in their drinking water [19]. These various studies linking PLG to immune cell migration led to a proteomics-based search for a migration-specific receptor for PLG, leading to the discovery and characterization of PLG-R_KT_ [20]. A PLG-R_KT_ monoclonal antibody inhibits invasion of monocytes in vitro into a matrigel in response to a chemotactic stimulus, MCP-1, and in vivo in a thioglycollate model [21], and this regulation of chemotactic migration appears specific to PLG-R_KT_ [20].

We show that PLG-R_KT_ is expressed broadly on many types of immune cells in our mouse line, including IP macrophages, blood monocytes, and PVMs. PVMs are replenished by bone marrow-derived cells [22] and are phagocytic cells that have a close association to the cerebral vasculature [23]. Because of their location in the perivascular space of blood vessels in the brain, PVMs are in an optimal location for interacting with circulating immune cells and cerebral endothelial cells and for communicating signals to the brain. There is also evidence that these cells may play a crucial role in maintaining homeostasis of the brain [23].

Previous studies with LPS have shown that sickness behavior occurs due to proinflammatory cytokines acting on the brain [5]. A recent study showed that bone marrow-derived PVMs play a crucial role in producing this proinflammatory effect in the brain; these cells release IL-1β as a response to LPS circulating in the blood in the subfornical area, one of the circumventricular organs that lacks a blood-brain barrier [24]. Our work supports the idea that PVMs are crucial for this neuroimmune response to systemic LPS challenge and also suggests that plasma PLG is crucial for the migration of PVMs into the brain.

**Fig. 4:**
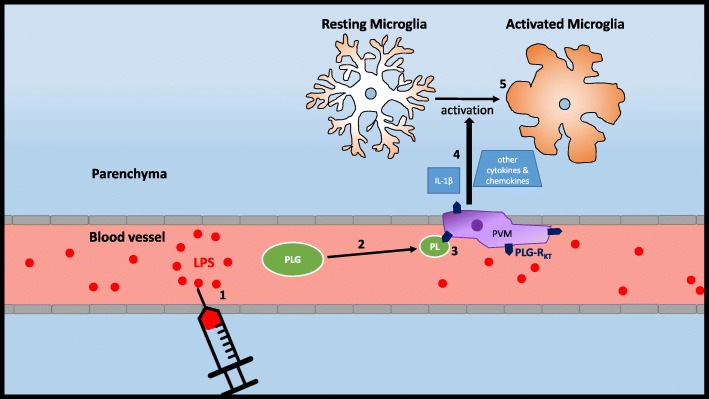
Proposed mechanism of how PLG mediates the systemic inflammatory and neuroinflammatory response during an LPS challenge. (1) LPS injected IP gets into the bloodstream of mice. (2) Inflammatory signals in the blood lead to PLG cleavage into plasmin. (3) Plasmin, which mediates macrophage migration, acts on PLG-R_KT_ receptors on PVMs. (4) PVMs release IL-1β and other cytokines and inflammatory mediators into the parenchyma of the brain which (5) causes activation of microglial cells

Although LPS is too large (50–100 kDa) to enter the brain parenchyma and directly activate microglia, PVMs exposed to LPS can penetrate the parenchyma and induce the release of interleukin 1β (IL-1β) and other cytokines, thereby leading to microglial activation and an astrocytic response. Although there is a full recovery of PVMs to PBS-treated CTRL ASO levels in mice treated with PLG ASO prior to LPS injection, activation of microglia/macrophages and astrocytes is still increased compared to controls, indicating that PVMs are not fully responsible for the neuroimmune response to LPS. For example, LPS can act directly on endothelial cells to stimulate the release of cytokines and other inflammatory mediators such as cyclooxygenase-2 into the parenchyma of the brain [10]. However, while it has been known that LPS causes activation of microglial cells, the mechanism remains elusive. Our current study increases evidence that PVMs likely play a crucial role in this response. A summary of our proposed mechanism is summarized in Fig. 4.

Notably, our data also suggest that plasma PLG depletion may be an effective treatment for neuroinflammatory conditions. However, PLG deficiency is known to cause other disorders, such as conjunctivitis of the eye, woody deposits on the gums, and gingivitis, all of which are linked to the inability to clear fibrin [16]. Because PLG has many physiological functions in the body, it may be challenging to just target those linked to chemotaxis and immunity, and thus it may not be the best therapeutic target for neuroinflammatory disease. It may be most effective to target the PLG-R_KT_ receptor to interfere with chemotaxis of immune cells like PVMs without affecting the overall PLG activity. The role PLG-R_KT_ may play in modulating neuroinflammation merits further studies to validate it as an important clinical target.

## Conclusions

The present study has shown that PLG is a key mediator of perivascular macrophage migration that plays a crucial role in the communication of inflammatory signals between the periphery and the CNS. While PLG depletion is likely not a valuable therapeutic strategy to treat or prevent neuroinflammatory conditions due to the pleiotropic effects of PLG in the body, targeting PLG-R_KT_, a key PLG-binding receptor for chemotaxis of monocytic cells, warrants further study.

## Data Availability

The authors should be contacted for any data and material requests.

## Abbreviations

AD: Alzheimer’s disease
ASO: Antisense oligonucleotide
CNS: Central nervous system
CTRL: Control
GFAP: Glial fibrillary acidic protein
IL-1β: Interleukin 1β
IP: Intraperitoneal
LPS: Lipopolysaccharide
PBS: Phosphate-buffered saline
PLG: Plasminogen
PVM: Perivascular macrophage
